# Deep-learning-based attenuation map generation in kidney single photon emission computed tomography

**DOI:** 10.1186/s40658-024-00686-4

**Published:** 2024-10-12

**Authors:** Kyounghyoun Kwon, Dongkyu Oh, Ji Hye Kim, Jihyung Yoo, Won Woo Lee

**Affiliations:** 1 Department of Health Science and Technology, Graduate School of Convergence Science and Technology, Seoul National University, Gwanggyo-ro 145, Yeongtong-gu, Suwon, Gyeonggi-do 16229 Republic of Korea; 2 Department of Nuclear Medicine, Seoul National University Bundang Hospital, 82 Gumi-ro, 173 Beon-gil, Bundang-gu, Seongnam, Gyeonggi-do 13620 Republic of Korea; 3 Department of Nuclear Medicine, Seoul National University College of Medicine, 103 Daehak-ro, Jongno-gu, Seoul, 03080 Republic of Korea; 4 Institute of Radiation Medicine, Medical Research Center, Seoul National University, 101 Daehak-ro, Jongno-gu, Seoul, 03080 Republic of Korea

**Keywords:** Attenuation correction, Deep learning, SPECT/CT, Kidney imaging, Quantitative imaging

## Abstract

**Background:**

Accurate attenuation correction (AC) is vital in nuclear medicine, particularly for quantitative single-photon emission computed tomography/computed tomography (SPECT/CT) imaging. This study aimed to establish a CT-free quantification technology in kidney SPECT imaging using deep learning to generate synthetic attenuation maps (μ-maps) from SPECT data, thereby reducing radiation exposure and eliminating the need for CT scans.

**Results:**

A dataset of 1000 Tc-99m DTPA SPECT/CT scans was analyzed for training (n = 800), validation (n = 100), and testing (n = 100) using a modified 3D U-Net for deep learning. The study investigated the use of primary emission and scattering SPECT data, normalization methods, loss function optimization, and up-sampling techniques for optimal μ-map generation. The problem of checkerboard artifacts, unique to μ-map generation from SPECT signals, and the effects of iodine contrast media were evaluated. The addition of scattering SPECT to primary emission SPECT imaging, logarithmic maximum normalization, the combination of absolute difference loss (L_1_) and three times the absolute gradient difference loss (3 × L_GDL_), and the nearest-neighbor interpolation significantly enhanced AI performance in μ-map generation (*p* < 0.00001). Checkerboard artifacts were effectively eliminated using the nearest-neighbor interpolation technique. The developed AI algorithm produced μ-maps neutral to the presence of iodine contrast and showed negligible contrast effects on quantitative SPECT measurement, such as glomerular filtration rate (GFR). The potential reduction in radiation exposure by transitioning to AI-based CT-free SPECT imaging ranges from 45.3 to 78.8%.

**Conclusion:**

The study successfully developed and optimized a deep learning algorithm for generating synthetic μ-maps in kidney SPECT images, demonstrating the potential to transition from conventional SPECT/CT to CT-free SPECT imaging for GFR measurement. This advancement represents a significant step towards enhancing patient safety and efficiency in nuclear medicine.

**Supplementary Information:**

The online version contains supplementary material available at 10.1186/s40658-024-00686-4.

## Background

Quantitative methods used in nuclear medicine rely heavily on accurate attenuation corrections (AC). The AC assesses the attenuation coefficients of the matter of interest. Initially, these coefficients were mathematically determined for homogeneous organs, such as the brain [1]. Later, transmission scans using external radionuclides generated attenuation maps (μ-maps) for non-homogeneous organs [2]. X-ray computed tomography (CT) has become the preferred method for AC across organs because of its superior image quality, reduced imaging time, and precise tissue delineation compared to radionuclide transmission scans [3].

Quantitative single-photon emission computed tomography/computed tomography (SPECT/CT) is an emerging modality in nuclear medicine, where CT plays a crucial role in AC [4]. The μ-map, based on CT Hounsfield units, integrates into the reconstruction of quantitative SPECT images. The results are clinically invaluable quantitative parameters, such as the percent injected dose (%ID) and standardized uptake value (SUV) of target organs [5–7].

Recent advancements in artificial intelligence (AI) have profoundly affected SPECT/CT imaging. Networks like convolutional neural network (CNN) or generative adversarial network (GAN) can create a synthetic μ-map without requiring a CT scan [8, 9]. These AI-driven innovations have been particularly effective in myocardial perfusion SPECT [10] and quantitative thyroid SPECT [11] imaging, leading to reduced radiation exposure in patients.

Technetium-99m diethylenetriaminepentaacetic acid (Tc-99m DTPA) renal scintigraphy is used to diagnose renal disorders because the renal uptake mechanism of Tc-99m DTPA is dependent on the glomerular filtration rate (GFR), a crucial biological measure of renal function [12]. A correlation between the GFR and %ID of Tc-99m DTPA has been established in multiple studies [13–15]. Although %ID measurement traditionally relied on 2-dimensional planar scintigraphy, 3-dimensional quantitative SPECT/CT imaging offers greater accuracy and consistency for renal %ID measurements and thus for GFR assessment [16].

Since 2017, our institution has been using Tc-99m DTPA SPECT/CT for GFR assessment [16]. We developed an automatic kidney segmentation technique using CNNs [17]. Our most recent development allows CNNs to generate a synthetic μ-map from SPECT data alone, eliminating the need for CT input, a method that has been successfully applied to CT-free thyroid SPECT imaging [11].

In this study, our primary objective was to establish a CT-free quantification methodology in kidney SPECT. By exclusively using SPECT data, we aimed to train the CNNs to create μ-maps. The ultimate goal was to transition from conventional glomerular filtration rate (GFR) SPECT/CT to CT-free GFR SPECT.

## Methods

### Dataset

Tc-99m DTPA SPECT/CT data from January 2022 to January 2023 were used in this study (Table 1), which retrospectively comprised 1000 SPECT/CT scans (male:female = 662:338, age 55.740 ± 12.967 years). Of these, 53.5% (535/1000) were performed immediately after iodine contrast-enhanced CT in the Radiology Department. This was primarily because the patients underwent oncologic evaluations with a primary focus on renal tumors. Consequently, varying amounts of iodine contrast remain in the urinary system during SPECT/CT imaging in the nuclear medicine department. These 1000 SPECT/CT images were allocated in an 8:1:1 ratio for training, validation, and testing. The proportions of contrast-enhanced CT scans were 54.0% (432/800), 53.0% (53/100), and 50.0% (50/100) for training, validation, and testing, respectively (Table 1). Details of the acquisition and reconstruction parameters are provided in the Supplementary Material.

**Table 1:** Characteristics of the datasets

		Training (n = 800)	Validation (n = 100)	Testing (n = 100)	*P* value
Gender (male:female)		531:269	66:34	66:34	0.9950
Age (years)		56.29 ± 12.87	54.50 ± 13.23	53.35 ± 13.27	0.0565
Height (cm)		166.08 ± 8.93	167.38 ± 9.24	168.57 ± 8.73	0.0303
Weight (kg)		69.24 ± 12.88	70.66 ± 12.65	73.28 ± 13.85	0.0217
BSA* (m^2^)		1.77 ± 0.19	1.79 ± 0.19	1.83 ± 0.20	0.0138
Proportion of contrast-enhanced CT		54.0% ( = 432/800)	53.0% ( = 53/100)	50.0% ( = 50/100)	0.7171
Reason for SPECT/CT	Normal (kidney donor)	37	2	1	0.0239
	Renal tumor	73	12	19	
	Urinary stone	126	20	9	
	Post partial nephrectomy	534	65	70	
	Post total nephrectomy	17	0	0	
	Hydronephrosis	8	0	0	
	Other	5	1	1	

^*^Body surface area by the Dubois formula: BSA (m2) = 0.007184 × (weight in kg)^0.425^ × (height in cm)^0.725^

Data are mean ± standard deviation

### Pre-processing for deep-learning

The AI algorithm was trained using SPECT scans (both primary emission and scattering) as inputs, with the CT-derived μ-map serving as the label. Here, the primary emission SPECT means the SPECT image reconstructed using primary photons at a 140 keV energy peak (20% window: 126–154 keV), while the scattering SPECT refers to the SPECT image reconstructed using scattered photons at a 120 keV energy peak (10% window: 115–125 keV). The primary emission and scattering SPECT images were reconstructed from the respective sinograms using vendor-provided software (Q. VolumetrixMI, GE Healthcare, Chicago, IL, USA) with correction for the collimator-detector response (i.e., resolution recovery, RR), resulting in NCRR SPECT. Here, NC indicates neither attenuation correction (AC) nor scatter correction (SC). Details of pre-processing are described in Supplemental Material.

### Network architecture

**Fig. 1:**
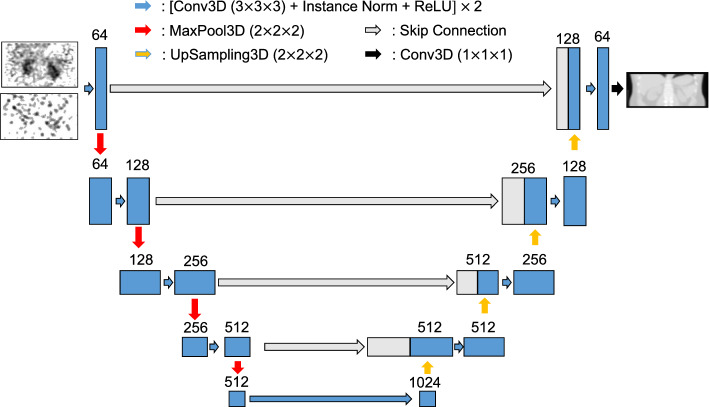
Network architecture for μ-map generation

A modified 3D U-net with 64 initial neurons and 4 skip connections was employed (Fig. 1) [11]. The modified architecture replaces batch normalization layer to instance normalization, and transpose convolution layer to nearest-neighbor interpolation. Details of network architecture are demonstrated in Supplemental Materials.

We trained our networks using TensorFlow [18] and the Keras framework [19].

### Loss function

The loss function for μ-map generator was defined as the combination of absolute difference loss (L_1_) and gradient difference loss (L_GDL_) with weighting factor ω.

$$L\left( {G\left( X \right),Y} \right) = L_{1} \left( {G\left( X \right),Y} \right) + \omega \times L_{GDL} \left( {G\left( X \right),Y} \right)$$

where G(X) represents generated synthetic μ-map from SPECT input X, and Y indicates ground truth of CT-derived μ-map. In this study, we did not consider the squared difference loss (that is, L_2_) because the superiority of L_1_ over L_2_ had been consistently reported in variable CT-related deep-learning studies such as those relating to the de-noising of low dose CT [20], reconstruction of micro CT [21], and μ-map generation from either positron emission tomography (PET) [22] or SPECT imaging [11]. However, L_GDL_ was investigated for absolute GDL (L_GDL_^1^) vs. squared GDL (L_GDL_^2^) (see Supplementary Material). The contested values of ω, the weighting factor of L_GDL_, were 1, 3, and 5.

### Training hyper-parameters

The number of training epochs was set to 100 with a batch size of eight. Early stopping rules were applied during the first 10 epochs. An adaptive moment estimation optimizer was used with a learning rate of 0.001 and an exponential decay rate of 0.96. Flip augmentation was applied along the x, y, and z axes. The training time was approximately 30 min per epoch. The computer hardware used for the network training was an AMD Ryzen7 5800X CPU (AMD Inc., Santa Clara, CA, USA) and an Nvidia RTX 3090 GPU (Nvidia Corp., Santa Clara, CA, USA).

### Metrics for outcome evaluations

To evaluate the performance of the AI algorithm for synthetic μ-map generation, R squared (R^2^), mean squared error (MSE), and percent normalized mean absolute error (%NMAE) were used for pixel-wise comparisons of attenuation coefficients.

*R*^2^, MSE, and %NMAE were defined as:

$$R^{2} = 1 - \frac{{\sum (\left( {G\left( X \right) - Y} \right)^{2} }}{{\sum \left( {Y - \overline{Y}} \right)^{2} }}$$

$${\text{MSE}} = \frac{1}{N}\sum \left( {G\left( X \right) - Y} \right)^{2}$$

$${\text{\% NMAE}} = \frac{1}{N} \cdot \frac{{\sum \left| {G\left( X \right) - Y} \right|}}{{\max \left( Y \right) - {\text{min}}\left( Y \right)}} \times 100{\text{\% }}$$

where N represents the total number of voxels as 1,048,576 ( = 64 × 128 × 128), Y the target (i.e., original μ-map), $\overline{Y}$ the mean of Y, and G(X) the synthetic μ-map from SPECT input X.

A kidney segmentation tool currently under development was applied to both conventional SPECT/CT and CT-free SPECT to measure the renal parenchymal radioactivity (manuscript in preparation). GFR was calculated from the parenchymal radioactivity (that is, %uptake) using the established equation: GFR (mL/min) = %uptake × 9.1462 + 23.0653 [16].

### Statistical analysis

Parametric tests (i.e., *t*-test or analysis of variance) were performed for continuous variables when the Shapiro-Wilk test did not reject normal distribution features. Otherwise, non-parametric tests (i.e., Mann-Whitney U test or Kruskal–Wallis test) were performed. Categorical variables were compared using the chi-squared test. The Friedman test was conducted to evaluate performance under optimal AI conditions. Statistical significance was set at *p* < 0.05. All analyses were performed using statistical software (MedCalc, version 22.013; Ostend, Belgium).

## Results

We investigated the optimal AI working conditions in generating μ-maps, starting from the following baseline conditions: using only primary emission SPECT as input, applying maximum normalization to the SPECT input, employing the L_1_ loss function, and utilizing transpose convolution in the expanding pathway of the CNN.

### Input spects

We first tested whether adding scattering SPECT to the primary emission SPECT would improve AI performance in μ-map generation. This is important because SPECT imaging has the advantage of easily obtaining scattering information, which is, in principle, difficult or impossible with PET imaging. The R^2^ value increased whereas the MSE and %NMAE values decreased, indicating an improvement in AI performance (P alone vs. PS, Table 2). These findings were actually consistent with previous results, which advocated for the combined use of SPECT inputs (primary emission and scattering SPECT scans) for the μ-map generation [8, 11].

**Table 2:** Performance of AI algorithms for synthetic μ-map generation (n = 100 testing cases)

Input	Normalization	Loss function	Up-sampling	R^2^	MSE (× 10^−4^)	%NMAE
P alone	Max	L_1_	TC	0.9802 ± 0.010300	1.1081 ± 0.5767	1.7975 ± 0.4452
PS	Max	L_1_	TC	0.9814 ± 0.009381	1.0417 ± 0.5367	1.7762 ± 0.4253
PS	Log-max	L_1_	TC	0.9818 ± 0.009789	1.0216 ± 0.5825	1.7049 ± 0.4588
PS	Log-max	L_1_+3 × L_GDL_^1^	TC	0.9822 ± 0.009555	0.9998 ± 0.5673	1.6790 ± 0.4315
PS	Log-max	L_1_+3 × L_GDL_^1^	Interpolation	0.9824 ± 0.009811	0.9880 ± 0.5601	1.6690 ± 0.4315
				*p* < 0.00001	*p* < 0.00001	*p* < 0.00001

P: primary emission SPECT, S: scattering SPECT, TC: transpose convolution, Max: maximum normalization, Log-max: logarithmic maximum normalization, Date are mean ± standard deviation

### Normalization of the input SPECT images

**Fig. 2:**
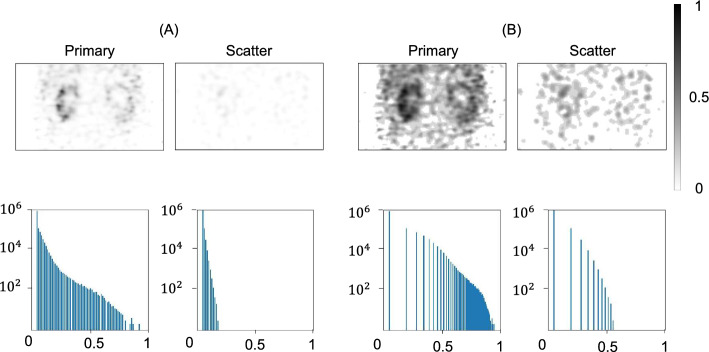
Effects of logarithmic maximum normalization on input single-photon emission computed tomography (SPECT) scans. **A** Maximum normalization of primary emission and scattering SPECT scans resulted in a highly asymmetric data distribution, exhibiting skewness values of 7.508 and 4.819 for primary emission and scattering SPECT scans, respectively (see the Supplementary Material for the equation of skewness). This approach also led to relatively weaker signal strength, with normalized mean attenuation coefficients of 0.028 cm^−1^ and 0.014 cm^−1^ for primary emission and scattering SPECT scans, respectively. **B** In contrast, logarithmic maximum normalization reduced the data imbalance, indicated by skewness values of 2.059 and 3.161 for primary emission and scattering SPECT scans, respectively. It also enhanced the signal strength, with normalized mean attenuation coefficients of 0.257 cm^−1^ and 0.162 cm^−1^ for primary emission and scattering SPECT scans, respectively.

Next, we investigated the normalization of the input SPECT images. This step was crucial because SPECT data often exhibit characteristics of localized SPECT signals in the renal parenchyma against very low background signals, leading to a highly asymmetric data distribution (Fig. 2). In this context, conventional maximum normalization could result in the under-representation of low signal areas in synthetic μ-map generation. Thus, we adopted a logarithmic maximum normalization method (see Supplemental Material) to mitigate the data scarcity or imbalance arising from the unique features of SPECT data. The application of logarithmic maximum normalization resulted in an overall greater signal strength and lower variance in the data distribution compared with conventional maximum normalization (Fig. 2). This approach was successful across all outcome measures of R^2^, MSE, and %NMAE (maximum vs. log-maximum, Table 2).

### Loss function optimization

Regarding the loss function, we tested the absolute GDL (L_GDL_^1^) versus the squared GDL (L_GDL_^2^) using three weighting factors (that is, 1, 3, and 5) in addition to the L_1_ loss function. L_GDL_^1^ with a weighting factor of three showed the best performance (Supplemental Table 1 and Supplemental Fig. 1). The change in the loss function from L_1_ alone to L_1_+3 × L_GDL_^1^ increased R^2^ and decreased the MSE and %NMAE (L_1_ vs. L_1_+3 × L_GDL_^1^, Table 2).

In a recent investigation, the L_GDL_ was tested without taking the absolute values of the operator [22] (supplemental equation); thus, we investigated different L_GDL_ with variable weighting factors but failed to show any benefit over the L_GDL_ using the absolute operator value (Supplemental Table 2).

### Nearest-neighbor interpolation during up-sampling

**Fig. 3:**
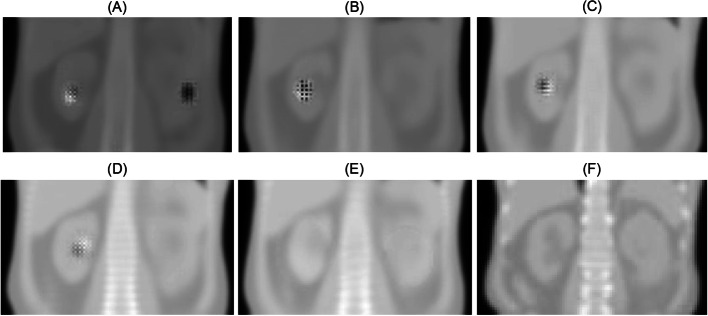
Resolution of checkerboard artefacts using optimal training conditions for μ-map generation. The sequence of applying training conditions follow the same order as presented in Table 2. **A** Shows the result using only primary emission single-photon emission computed tomography (SPECT) as input. **B** depicts the outcome with the addition of scattering SPECT to primary emission SPECT (PS) imaging. **C** illustrates the effect of applying log-max normalization to the PS input. **D** presents the result using the additional 3 × L_GDL_^1^ loss function to **C**. **E** demonstrates the impact of applying nearest-neighbor interpolation to **D**, replacing transpose convolution. **F** provides the ground truth of the CT-derived μ-map for comparison

**Fig. 4:**
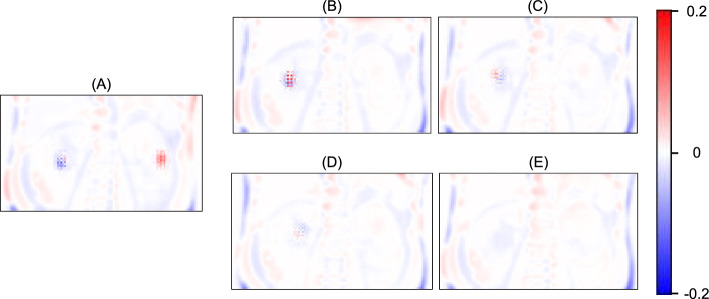
Error maps showing the resolution of checkerboard artifacts. Individual panels show the difference of attenuation coefficients between the ground truth (computed tomography-derived μ-map) vs. **A** primary emission single-photon emission computed tomography (SPECT) alone input, **B** primary emission and scattering SPECT (PS) input, **C** PS with log-max normalization, **D** PS with log-max normalization using an additional 3 × L_GDL_^1^ loss function, and **E** nearest-neighbor interpolation application to **D** instead of transpose convolution. The scale bar indicates the attenuation coefficients in unit of cm^−1^

**Fig. 5:**
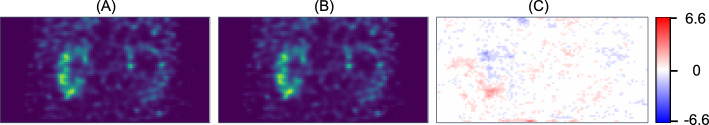
Comparison of ASCSRR (attenuation correction, scatter correction, and resolution recovery) SPECT images between the AI-based corrected SPECT **A** and the ground truth CT-based corrected SPECT **B**. The differences were minimal in terms of SPECT counts/voxel **C**

The SPECT input sometimes exhibited high focal activity in the kidney, which often resulted in checkerboard artifacts in the synthetic μ-map (Fig. 3A). Such artifacts have been reported to occur in or adjacent to high-signal areas during image generation using neural networks [23]. In the case of kidney SPECT imaging, artifacts often appeared in both kidneys when primary emission SPECT was used alone as input (Fig. 3A), and tended to lateralize to one of the two kidneys when scattering SPECT was additionally employed as input (Fig. 3B). Application of log-max normalization slightly diminished the size of the artifacts (Fig. 3C). Moreover, the addition of a 3 × L_GDL_^1^ loss function systemically shifted the artifact location from the renal parenchyma to the central renal pelvis (Fig. 3D). Finally, replacing the transpose convolution with nearest-neighbor interpolation completely eliminated checkerboard artifacts (Fig. 3E), thereby improving the outcome parameters R^2^, MSE, and %NMAE (TC vs. interpolation, Table 2). The error maps of attenuation coefficients (the ground truth minus the generated synthetic μ-maps) clearly demonstrated the sequential reduction of the checkerboard artifact (Fig. 4). As a result, the AI-based corrected ASCSRR SPECT was indistinguishable from the ground truth CT-based corrected ASCSRR SPECT, with minimal differences (Fig. 5).

The interpolation application was also tested in various ways, but modifications of the nearest-neighbor interpolation produced halo artifacts (Supplemental Table 3 and Supplemental Fig. 2); thus, further modifications were abandoned.

**Table 3:** Performance of AI algorithms for synthetic μ-map generation in SPECT/CT cases with iodine contrast effect (n = 50)

Input	Normalization	Loss function	Up-sampling	R^2^	MSE (× 10^−4^)	%NMAE
P alone	Max	L_1_	TC	0.9801 ± 0.009098	1.1249 ± 0.500390	1.8288 ± 0.404589
PS	Max	L_1_	TC	0.9818 ± 0.008471	1.0316 ± 0.470583	1.7831 ± 0.374997
PS	Log-max	L_1_	TC	0.9818 ± 0.008833	1.0374 ± 0.504221	1.7323 ± 0.407806
PS	Log-max	L_1_+3 × L_GDL_^1^	TC	0.9822 ± 0.008021	1.0010 ± 0.458064	1.6994 ± 0.364543
PS	Log-max	L_1_+3 × L_GDL_^1^	Interpolation	0.9827 ± 0.007784	0.9821 ± 0.436738	1.6780 ± 0.361764

P: primary emission SPECT, S: scattering SPECT, TC: transpose convolution, Max: maximum normalization, Log-max: logarithmic maximum normalization, Date are mean ± standard deviation

### The iodine contrast effects for μ-map generation

We divided the 100 SPECT/CT cases in the testing group into 50 cases with contrast effects from the previous iodine contrast-enhanced CT (Table 3) and 50 cases without these effects (Table 4). Overall, the same trends in AI performance improvement were observed in both subgroups using optimal training conditions for AI (that is., PS [primary emission and scattering SPECT imaging]) input, logarithmic maximum normalization of the SPECT images, L_1_ plus 3 × L_GDL_^1^ combinatory loss function, and nearest-neighbor interpolation during up-sampling). One exception was found in the last step of the subgroup without the iodine contrast effect (Table 4). Here, the application of nearest-neighbor interpolation instead of transpose convolution slightly reduced R^2^ and increased the MSE and %NMAE (Table 4). However, only when using the nearest-neighbor interpolation did the checkerboard artifacts completely disappear (Fig. 3E for μ-map, and Fig. 4E for error map of attenuation coefficients).

**Table 4:** Performance of AI algorithms for synthetic μ-map generation in SPECT/CT cases without iodine contrast effect (n = 50)

Input	Normalization	Loss function	Up-sampling	R^2^	MSE (× 10^−4^)	%NMAE
P alone	Max	L_1_	TC	0.9802 ± 0.011486	1.0912 ± 0.648855	1.7663 ± 0.484568
PS	Max	L_1_	TC	0.9809 ± 0.010364	1.0517 ± 0.600247	1.7693 ± 0.474100
PS	Log-max	L_1_	TC	0.9819 ± 0.010758	1.0058 ± 0.656376	1.6776 ± 0.507255
PS	Log-max	L_1_+3 × L_GDL_^1^	TC	0.9821 ± 0.010999	0.9901 ± 0.663409	1.6586 ± 0.492409
PS	Log-max	L_1_+3 × L_GDL_^1^	Interpolation	0.9820 ± 0.011443	0.9938 ± 0.665576	1.6600 ± 0.495213

P: primary emission SPECT, S: scattering SPECT, TC: transpose convolution, Max: maximum normalization, Log-max: logarithmic maximum normalization, Date are mean ± standard deviation

**Fig. 6:**
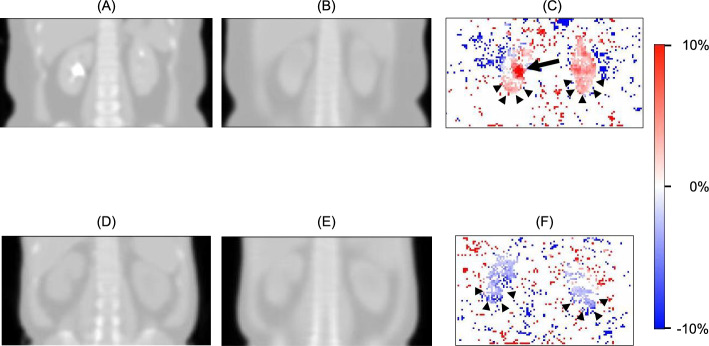
Characteristics of the artificial intelligence (AI) algorithm neutral to the iodine contrast media. **A** and **D** represent the ground truth μ-maps with and without iodine contrast media effects, respectively. **B** and **E** are the corresponding synthetic μ-maps generated by the AI algorithm. **C** and **F** show the corresponding error maps of radioactivity, comparing the ground truth single-photon emission computed tomography/CT (SPECT/CT) with CT-free SPECT imaging. Despite the presence of contrast-media in the renal pelvis of the CT-driven μ-map **A**, the synthetic μ-map generated by the AI algorithm did not exhibit contrast effects **B**. This can be appreciated in the intense red area of the right renal pelvis on the error map of radioactivity (long arrow) **C**. The μ-map with iodine contrast in the renal parenchyma, while subtle in the ground truth μ-map **A**, had slightly higher attenuation coefficients than the AI-driven synthetic μ-map **B**, as evident in the error map (arrow heads) **C**. The ground truth μ-map without iodine contrast **D** showed slightly lower attenuation coefficients in the renal parenchyma than the AI-driven synthetic μ-map **E**, which was noticeable in the error map (arrow heads) **F**

The AI algorithm was trained on approximately half the cases involving contrast-enhanced CT as labels (Table 1). In other words, the iodine contrast media was present in half of the cases of the ground truth μ-maps during the AI training. The existence of iodine contrast media was typically apparent in the renal pelvis (Fig. 6A), but the trained AI algorithm consistently generated μ-maps without iodine contrast effects in the renal pelvis (Fig. 6B). This was because the μ-maps were primarily created from SPECT radioactivity signals originating from the renal parenchyma. The renal pelvis is the site of urine accumulation without a functional renal parenchyma. As a result, the output of the AI algorithm was always a signal void in the renal pelvis, even in the presence of iodine contrast medium in the ground truth (Fig. 6B). In contrast, the presence of iodine contrast media in the renal parenchyma was visually inconspicuous (Fig. 6A) and led to a subtle increase in attenuation coefficients compared to the synthetic μ-map (Fig. 6B), subsequently leading to over-correction of radioactivity in the renal parenchyma (arrow heads in Fig. 6C). The levels of attenuation coefficients in the synthetic μ-maps fell within an intermediate range between those derived from contrast-enhanced and non-contrast-enhanced CT, which was indicated by the positive difference from the contrast-enhanced ground truth and the negative difference from the non-contrast-enhanced ground truth (Table 5). Therefore, the radioactivity associated with CT-free SPECT and the subsequent GFR values derived from the developed AI algorithm were significantly lower (*p* < 0.0001) than those obtained by SPECT/CT with contrast effects (n = 50), but significantly higher (*p* < 0.0001) than those obtained by SPECT/CT imaging without contrast effects (n = 50; Table 5). However, the effect of the presence of contrast media was insignificant because the maximum difference in GFR using the extreme limits for contrast CT (0.4530 + 1.0658 mL/min = 1.5188 mL/min) and non-contrast CT (−0.4394–0.9316 mL/min = −1.371 mL/min) was only 2.8898 mL/min, which was only 2.78% of the mean GFR values in this study (Table 5). Furthermore, in a total of 100 SPECT/CT cases collectively, the sums of attenuation coefficients (1147.0796 ± 216.4121 cm^−1^ vs. 1146.5204 ± 215.2922 cm^−1^), the quantitative radioactivity (6.9259 ± 1.6659% vs. 6.9232 ± 1.6600%), and the GFR values (104.4920 ± 17.1096 mL/min vs. 104.4852 ± 17.1736 mL/min) in the renal parenchyma were not significantly different between the ground truth SPECT/CT and the AI-driven CT-free SPECT (*p* > 0.05). Therefore, all of the findings above can be attributed to the nearly equal contribution of contrast-enhanced and non-contrast-enhanced CT to the development of the AI algorithm for μ-map generation (Table 1).

**Table 5:** The difference between the SPECT/CT (the ground truth) and the AI-driven CT-free SPECT imaging according to the presence of iodine contrast media in the SPECT/CT imaging (n = 100)

	Presence of contrast media (n = 50)	No contrast media (n = 50)	*P* value
Attenuation coefficients (cm^−1^)	19.8139 ± 15.7953	−18.8138 ± 5.9526	< 0.0001
Radioactivity (%point)	0.0517 ± 0.1167	−0.0464 ± 0.1058	< 0.0001
GFR (mL/min)	0.4530 ± 1.0658 (99.7065 ± 16.2896 vs. 99.2535 ± 15.9208 for SPECT/CT vs. CT-free SPECT, respectively)	−0.4394 ± 0.9316 (109.2775 ± 16.7141 vs. 109.7169 ± 16.9279 for SPECT/CT vs. CT-free SPECT, respectively)	< 0.0001

### The reduction of radiation exposure to patients

Radiation exposure from SPECT/CT, which included both SPECT and CT components, ranged from 3.313 to 8.563 mSv in terms of effective dose. The SPECT component from the Tc-99m DTPA injection was 0.0049 mSv/MBq [24], resulting in an effective dose of 1.813 mSv for a 370 MBq injection. The effective dose from CT component varied within a range of 1.5–6.75 mSv, using a conversion factor of 15 μSv/mGy-cm [25]. This variation was due to the variable dose-length product (100–450 mGy-cm), reflecting the differing extents of CT coverage of the abdomen and pelvis. Consequently, the potential reduction in radiation exposure by transitioning from conventional SPECT/CT to AI-based CT-free SPECT is 45.3–78.8%.

## Discussion

The issue of AC in nuclear medicine, specifically SPECT imaging, has been effectively addressed over the last few decades with the advent of hybrid scanners, such as SPECT/CT scanners [2, 3]. Despite the widespread adoption of these scanners, concerns regarding radiation exposure in patients have persisted in the field of nuclear medicine. Moreover, misalignment between CT and SPECT images, often resulting from patient motion, has been a significant topic of discussion regarding the accuracy of quantitative SPECT/CT [26]. Consequently, alternatives to CT have been intensively explored.

Recent advancements in AI have shown promise for various medical imaging applications, including reducing radiation exposure to patients and enabling CT-less imaging. These studies required the acquisition of low-dose or ultra-low-dose CT, allowing the enhancement of CT images with poor signal-to-noise ratios to the quality of usual dose CT through AI applications [27, 28]. Several other researchers have focused primarily on AI-driven AC techniques for SPECT. Those studies did not require CT acquisition, and the performance of the AI was validated using SPECT/CT as a reference, paving the way for CT-free (rather than CT-less) imaging studies [8, 29, 30].

In this study, we explored the potential use of AI as a substitute for CT in the AC of kidney SPECT. Our approach was inspired by other studies on myocardial perfusion SPECT/CT [9, 31] or thyroid SPECT/CT imaging [11]. In these studies, AI algorithms were trained using only SPECT images as inputs and CT-derived μ-maps as labels, thus generating synthetic μ-maps for AC of SPECT imaging. We optimized the AI working conditions (incorporating scattering SPECT with primary emission SPECT as input, applying log-maximum normalization instead of maximum normalization for SPECT input, using a combined loss function of L_1_ and 3 × L_GDL_^1^ and preferring nearest-neighbor interpolation over transpose convolution in the CNN up-sampling process) and demonstrated the effectiveness of the developed AI algorithm for Tc-99m DTPA kidney SPECT.

Previous deep-learning-based studies on AC in nuclear medicine imaging have primarily focused on PET rather than SPECT [32–34]. We believe that a completely different approach may be required for SPECT because scattering information is more readily obtainable for SPECT than for PET scans, and the addition of scattering SPECT consistently improved the performance of AI algorithms for the μ-map generation in other SPECT studies [8, 9, 11]. The standard OSEM reconstruction algorithm may be effective for single-bed SPECT applications such as kidney SPECT or thyroid SPECT [11]. This contrasts with PET, which typically requires whole-body coverage and a more complicated reconstruction algorithm [22, 35].

## Limitations

Despite the promising outcomes of our study, it is important to acknowledge its limitations. Firstly, the study utilized an 8:1:1 allocation for training, validation, and test sets, respectively. While the dataset included 1000 cases, the relatively smaller proportions for validation and test sets may have impacted the model’s reliability. This aspect warrants further investigation in future research. Secondly, our study was conducted within a single institution, which might introduce a selection bias and limit the external validity of the findings. Thirdly, while our AI algorithm showed high accuracy in generating μ-maps neutral to contrast-media effects, it was trained and tested on datasets from specific CT and SPECT machines. The performance of the algorithm might vary when applied to data from different equipment or settings, necessitating additional tuning and validation. Fourthly, our study focused primarily on kidney SPECT/CT imaging for GFR measurement; thus, the application of our findings to other organs or conditions requires further investigation. Finally, the impact of the AI algorithm on clinical outcomes was not directly assessed in this study. Future research should aim to not only replicate these findings in a multi-center context but also explore the clinical implications of using AI-supported CT-free SPECT imaging in routine practice.

## Conclusion

We clarified the importance of scattering information for μ-map generation in SPECT, found the effect of logarithmic maximum normalization on the input SPECTs, optimized the loss function and removed SPECT-specific checkerboard artifacts by an interpolation up-sampling. The AI algorithm was influenced equally by both contrast-enhanced and non-contrast-enhanced CT scans. As a result, it generated μ-maps with attenuation coefficients in an intermediate range, making the CT-free SPECT imaging neutral to the effects of contrast-media present in the ground truth SPECT/CT. Conventional kidney SPECT/CT imaging for GFR measurement could potentially be replaced by CT-free SPECT imaging using the developed AI algorithm.

## Supplementary information

Supplementary Material

## Data Availability

The datasets generated and/or analysed during the current study are not publicly available due to restrictions imposed by IRB (Institutional Review Board) but are available from the corresponding author on reasonable request.
