# Supplementary material for: Deep-learning-based attenuation map generation in kidney single photon emission computed tomography
Source: EJNMMI Phys. 2024 Oct 12;11:84. doi: 10.1186/s40658-024-00686-4 (PMC11469987; doi:10.1186/s40658-024-00686-4)

**Acquisition and reconstruction of kidney SPECT/CT**

No dietary restrictions were imposed during the acquisition of the Tc-99m DTPA kidney SPECT/CT imaging. Half an hour before the SPECT/CT imaging, 500 mL of water was provided to the patients for hydration. Patients were positioned on the table of one of two dual-head SPECT/CT scanners (NMCT670 or NMCT670pro; GE Healthcare, Chicago, IL, USA) equipped with low-energy high-resolution collimators. Tc-99m DTPA (TechneScan^R^ DTPA; Mallinckrodt Pharmaceuticals, Dublin, Ireland) was intravenously injected via the antecubital vein at an activity of 370 MBq.

SPECT images were acquired–2-3 minutes post-injection of Tc-99m DTPA under the following conditions: primary emission energy at 140 keV (20% window: 126-154 KeV), scatter energy at 120 keV (10% window: 115-125 KeV), 1-minute continuous acquisition mode with counter-clockwise rotation, no body contour option, and acquisition zoom factor of 1.28. Immediately after the SPECT scan, a helical CT scan was performed using the following parameters: tube voltage of 120 KVp, tube current of 60-210 mA with autoMa function at a noise level of 20, detector collimation of 20 mm (= 16$\times$1.25 mm), helical thickness of 2.5 mm, table speed of 37 mm/sec, table feed per rotation of 18.75 mm/rot, tube rotation time of 0.5 sec, and pitch of 0.938:1.

SPECT reconstruction was performed using iterative ordered-subset expectation–maximization (OSEM) (4 iterations and 10 subsets; Q.Metrix or Q.VolumetrixMI, GE Healthcare, Chicago, IL, USA). Attenuation correction (AC), scatter correction (SC), and resolution recovery (RR) were applied to the reconstruction of the quantitative SPECT (ACSCRR SPECT). Post-reconstruction Butterworth filter was used with cutoff frequency of 0.48 cycles/cm and order of 10. SPECT image matrix was 128x128x128 and voxel size was 3.45$\times$3.45$\times$3.45 mm^3^. CT images were reconstructed into a 512 × 512 × 161 matrix and 0.977 × 0.977 × 2.5 mm^3^ voxel size.

**Details of pre-processing**

The ordered subset expectation-maximization (OSEM) algorithm was used for SPECT reconstruction with 10 subsets and four iterations. For statistical noise reduction, a Butterworth low-pass filter (order of 10 and cutoff frequency of 0.48 cycles/cm) was applied to the scattering SPECT images. The ground truth μ-map was derived from the CT component of SPECT/CT using the software (Q.VolumetrixMI, GE Healthcare, Chicago, IL, USA). The original matrix and voxel sizes for both SPECT images and the μ-map were 128$\times$128$\times$128 and 3.45$\times$3.45$\times$3.45 mm^3^, respectively. All images were cropped to 64$\times$128$\times$128 to shorten the z-axis span, which was intended to ensure consistency in the training volumes along the long body axis (z-axis).

**Details of architecture**

The network architecture comprised contraction and expansion paths with skip connections between the two paths. During the contraction path, convolution blocks consisting of a 3 × 3 × 3 kernel, instance normalization, and rectified linear unit (ReLU) activation were consecutively used twice. Subsequently, 2 × 2 × 2 max pooling was used for downsampling. The convolution and max-pooling layers were consecutively applied 4 times, followed by an expansion path.

In the expansion path for up-sampling, nearest-neighbor interpolation and 2 times of the same convolution blocks (3 × 3 × 3 kernel, instance normalization, and ReLU activation) were consecutively applied four times. Finally, a 1 × 1 × 1 kernel convolution layer was applied without an activation function.

**Loss functions**

The L_1_ loss function was defined as:

$$L_{1}=\frac{1}{N}\sum\left| G\left( X \right)-Y \right|$$

where N was the total number of voxels in the μ-map as a fixed value of 1,048,576 (= 64×128×128).

The gradient difference loss (L_GDL_) was defined as:

$${L_{GDL}}^{n}=\frac{1}{M}\sum\left| \left| \nabla G(X) \right|-\left| \nabla Y \right| \right|^{n}$$

where M was the product of total number of voxels and the number of axes for gradients in the μ-map (3,145,728= 64×128×128×3), and ∇ was the image gradient operator. Here, n was either 1 for the absolute GDL (L_GDL_^1^) or 2 for the squared GDL (L_GDL_^2^).

**The equations for normalization**

The equation for the maximum normalization is:

$$V_{norm}=\frac{V_{origin}-V_{min}}{V_{max}-V_{min}}$$

The equation for the logarithmic maximum normalization is:

$$V_{norm}=\frac{{log}_{10}(1+V_{origin}-V_{min})}{{log}_{10}(1+V_{max}-V_{min})}$$

where V_norm_, V_origin_, V_max_ and V_min_ represent the voxel values for the normalized, original, maximum, and minimum values, respectively.

**The definition of skewness**

The skewness (S) was calculated using the following equation:

$$S=\frac{1}{N}\sum\frac{\left( V-V_{mean} \right)^{3}}{{std}^{3}}$$

where N is degree of freedom in a given image (= 64×128×128-1), V voxel value,$V_{mean}$ mean voxel value, and std standard deviation of V.

Supplemental Table 1. Tests of gradient difference losses with variable weighting factors in addition to the L_1_ loss function (n=100)

| Input | Normalization | Loss function | Up-sampling | R^2^ | MSE ($\times$10^-4^) | %NMAE |
| --- | --- | --- | --- | --- | --- | --- |
| PS | Log-max | L_1_+1$\times$L_GDL_^1^ | TC | 0.9817±0.010076 | 1.0252±0.595455 | 1.7019±0.459276 |
| PS | Log-max | L_1_+1$\times$L_GDL_^2^ | TC | 0.9812±0.009678 | 1.0523±0.557860 | 1.7409±0.425118 |
| PS | Log-max | L_1_+3$\times$L_GDL_^1^ | TC | 0.9822±0.009578 | 0.9998±0.567257 | 1.6790±0.431515 |
| PS | Log-max | L_1_+3$\times$L_GDL_^2^ | TC | 0.9810±0.010122 | 1.0676±0.587252 | 1.7369±0.446755 |
| PS | Log-max | L_1_+5$\times$L_GDL_^1^ | TC | 0.9819±0.009845 | 1.0153±0.569324 | 1.7131±0.428341 |
| PS | Log-max | L_1_+5$\times$L_GDL_^2^ | TC | 0.9817±0.009332 | 1.0271±0.548956 | 1.6995±0.430501 |

Supplemental Figure 1. Tests for Weighting Factors of L_GDL_. Upper row indicates MSE ($\times$10^-4^), while lower row %NMAE. Weighting factor 3 with absolute GDL loss (3$\times$L_GDL_^1^) showed the lowest MSE and %NMAE.


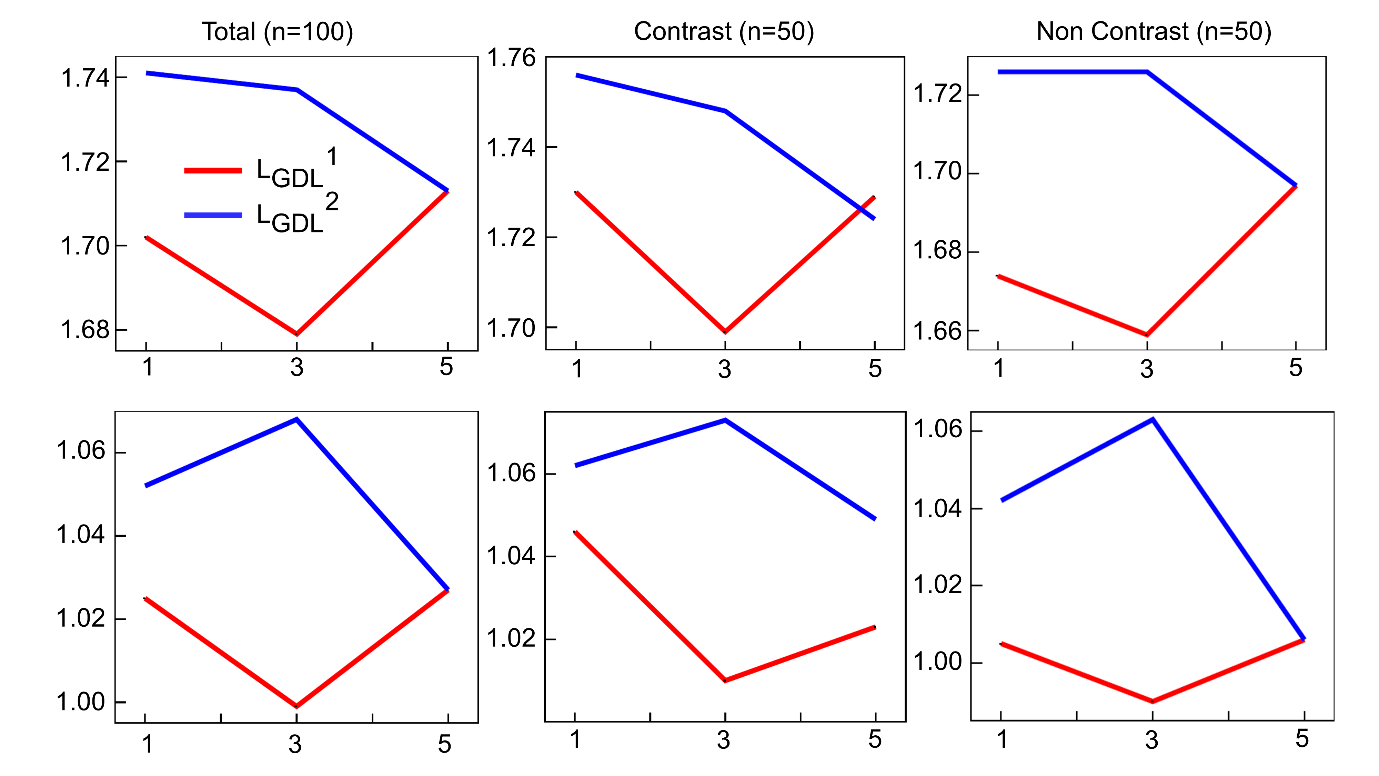


Supplemental Table 2. Other tests of gradient difference losses without taking the absolute operator values (n=100)

| Input | Normalization | Loss function | Up-sampling | R^2^ | MSE ($\times$10^-4^) | %NMAE |
| --- | --- | --- | --- | --- | --- | --- |
| PS | Log-max | L_1_+0.3$\times$L_GDL_^1^ | TC | 0.9819±0.009488 | 1.0151±0.566094 | 1.7029±0.443889 |
| PS | Log-max | L_1_+0.3$\times$L_GDL_^2^ | TC | 0.9815±0.009740 | 1.0351±0.566510 | 1.7060±0.428048 |
| PS | Log-max | L_1_+1$\times$L_GDL_^1^ | TC | 0.9817±0.010493 | 1.0221±0.597001 | 1.7048±0.438370 |
| PS | Log-max | L_1_+1$\times$L_GDL_^2^ | TC | 0.9812±0.009688 | 1.0535±0.564586 | 1.7307±0.439622 |
| PS | Log-max | L_1_+3$\times$L_GDL_^1^ | TC | 0.9812±0.009531 | 1.0539±0.559530 | 1.7374±0.438099 |
| PS | Log-max | L_1_+3$\times$L_GDL_^2^ | TC | 0.9818±0.009172 | 1.0231±0.537567 | 1.6977±0.427247 |

The gradient difference loss (L_GDL_) was calculated using the operator without taking absolute values, as follows:

$${L_{GDL}}^{n}=\frac{1}{M}\sum\left| \nabla G(X)-\nabla Y \right|^{n}$$

where M was the product of total number of voxels and the number of axes for gradients in the μ-map (3,145,728= 64×128×128×3), and ∇ was the image gradient operator. Here, n was either 1 for the absolute GDL (L_GDL_^1^) or 2 for the squared GDL (L_GDL_^2^). The weighting factors investigated in these tests were 0.3, 1, and 3.

Supplemental Table 3. Tests of different interpolations for up-sampling (n=100)

| Input | Normalization | Loss function | Up-sampling | R^2^ | MSE ($\times$10^-4^) | %NMAE |
| --- | --- | --- | --- | --- | --- | --- |
| PS | Log-max | L_1_+3$\times$L_GDL_^1^ | Interpolation+convolution (2$\times$2$\times$2 kernel) | 0.9827±0.010000 | 0.9695±0.582834 | 1.6858±0.436584 |
| PS | Log-ma$\times$ | L_1_+3$\times$L_GDL_^1^ | Interpolation+convolution block* | 0.9827±0.010142 | 0.9701±0.580632 | 1.6638±0.435304 |

*The convolution block consists of a 2 × 2 × 2 kernel, an instance normalization layer, and ReLU activation.

Supplemental Figure 2. Artifacts caused by modification of nearest-neighbor interpolation. (A) nearest-neighbor interpolation only, (B) nearest-neighbor interpolation plus 2 × 2 × 2 kernel convolution, and (C) nearest-neighbor interpolation plus a convolution block (2 × 2 × 2 kernel, instance normalization, and ReLU activation). Please see the red arrows for the halo artifacts in (B) and (C).


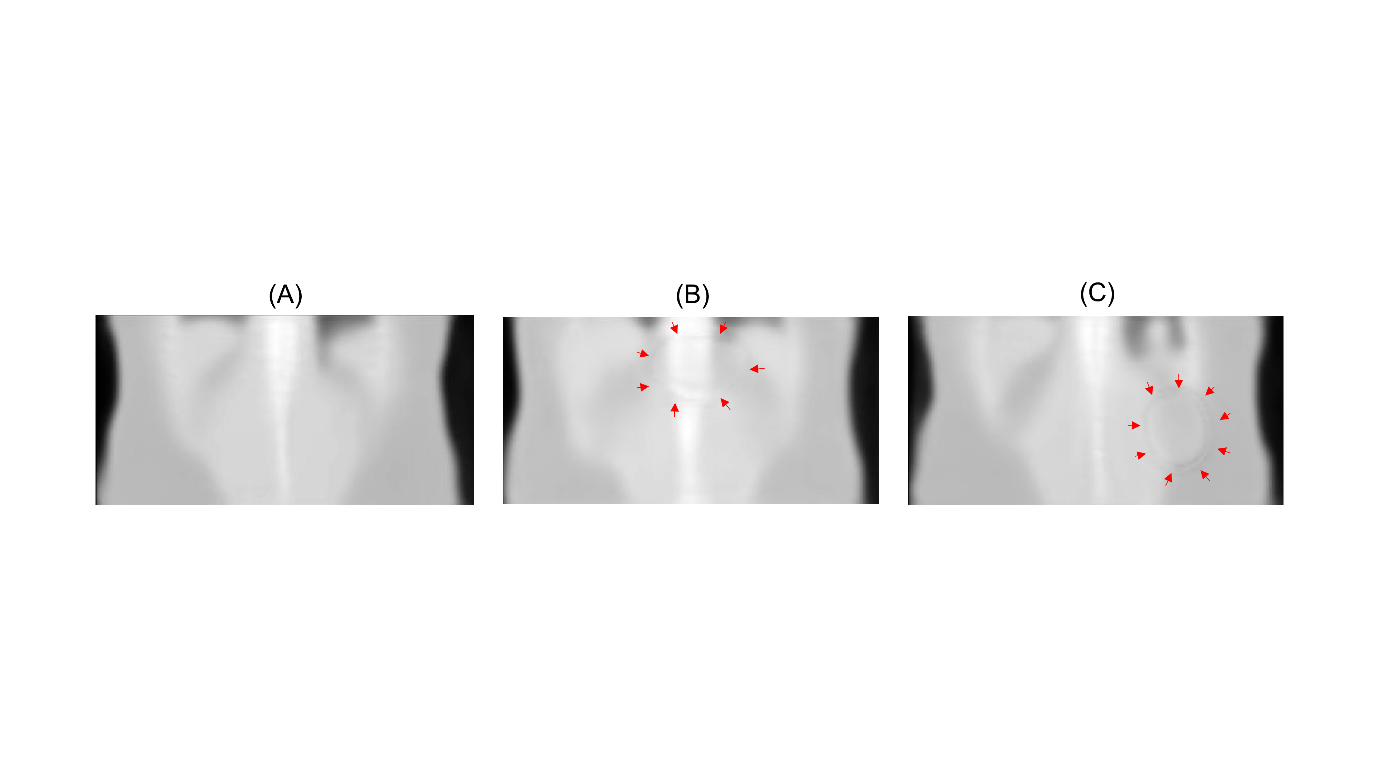

Supplement: Supplementary file 1 — Supplementary Material [file 40658_2024_686_MOESM1_ESM.docx]
